# Levetiracetam-Induced Rhabdomyolysis Following Medication Re-Initiation

**DOI:** 10.7759/cureus.30042

**Published:** 2022-10-07

**Authors:** Klayme-Shane Boucher, Neilay Dedhia, Deepak Bommisetty

**Affiliations:** 1 Internal Medicine, University of Texas at San Antonio, San Antonio, USA; 2 Internal Medicine, Methodist Health System, Dallas, USA

**Keywords:** seizure medications, keppra, antiepileptic, creatine kinase, adverse effect, medication re-initiation side effect, rhabdomyolysis, levetiracetam

## Abstract

Rhabdomyolysis is a potentially life-threatening condition in which skeletal muscle breaks down, resulting in the release of myoglobin and creatine kinase (CK) in the blood; CK accumulation can lead to kidney failure and death. Several case reports have reported incidences of levetiracetam (LEV)-induced rhabdomyolysis. However, there are currently no reports of new-onset rhabdomyolysis after restarting LEV in a patient who previously tolerated the medication with no side effects.

In this report, we present the case of a 35-year-old male who developed rhabdomyolysis after being restarted on LEV following a generalized tonic-clonic seizure. The patient was loaded with LEV 1 g IV and subsequently restarted on LEV 500 mg PO BID immediately after admission, from which time his serum CK level began to steadily rise to a maximum of 47,078 U/L despite aggressive intravenous hydration. LEV was discontinued on day five of admission when it was suspected to be the cause of the elevated CK levels in the absence of other contributing factors. The patient’s CK level decreased to 35,635 U/L on day six of admission and continued to decrease before reaching 5,556 U/L at discharge.

It is important to closely monitor serum CK in patients initiating or restarting LEV. Other antiepileptic medications should be considered if CK levels remain persistently elevated without other inciting factors.

## Introduction

Levetiracetam (LEV) is a common antiepileptic medication used alone or in combination with other antiepileptic medications to treat generalized tonic-clonic, myoclonic, and partial-onset seizures. LEV has been reported to have fewer and less severe side effects than other antiepileptic medications. Side effects commonly reported in LEV tolerability trials include headaches, nausea, dizziness, somnolence, fatigue, asthma, and irritability [1, 2]. In the literature, there are only a few reported cases of LEV-associated rhabdomyolysis and no reports of new-onset rhabdomyolysis after restarting LEV in a patient who had previously tolerated the medication with no adverse effects [3]. Rhabdomyolysis is a syndrome involving necrosis of skeletal muscle cells and the release of cellular contents such as myoglobin and creatine kinase (CK) into the blood. Here, we report the case of a patient who developed persistently elevated CK levels due to rhabdomyolysis following re-initiation of LEV after a month of medication non-adherence for generalized tonic-clonic seizures.

## Case presentation

**Figure 1:**
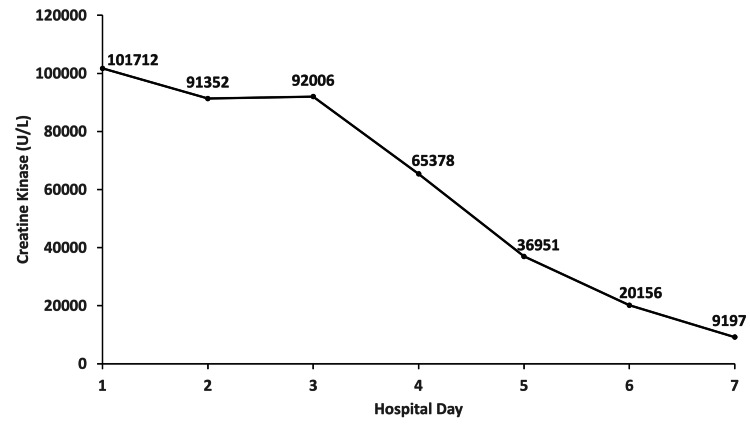
Serum creatine kinase trends from the patient’s previous hospital admission, at which time LEV was initiated on hospital Day 1.

A 35-year-old male with past medical history significant for generalized seizures and an intracranial hemorrhage due to trauma six years prior presented to the emergency department (ED) for evaluation and management of an unwitnessed seizure the night prior to admission. The patient had been placed on LEV during a previous hospitalization six months prior for a seizure where he had a CK of 101,712 U/L on admission, was started on LEV on hospital Day 1, and had downward trending CK levels throughout his hospital course (Figure 1); he had no adverse effects to the LEV at this time.

**Figure 2:**
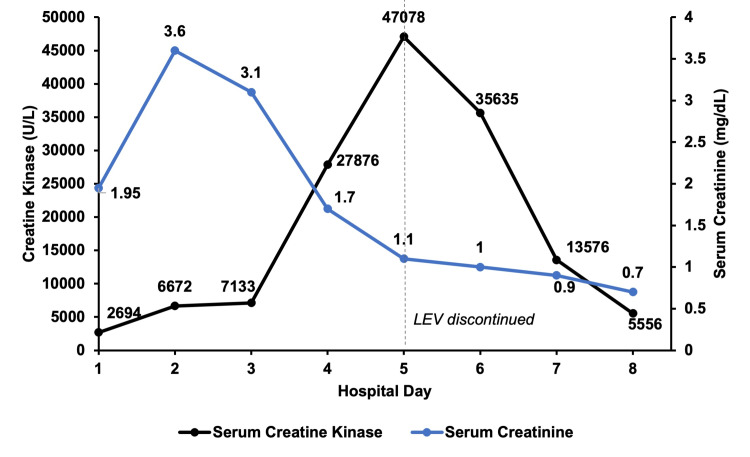
Serum creatine kinase and creatinine trends during the current hospitalization. Levetiracetam (LEV) was started on Day 1 of admission and halted on Day 5.

At the current presentation, he reported that he had stopped taking LEV a month prior. In the ED, the patient was alert and oriented and his neurological exam was unremarkable. He was given IV fluids, loaded with LEV 1g IV, and admitted for further monitoring and management. Neurology was consulted and LEV 500 mg PO BID was resumed given the patient’s history of generalized seizures. Neurology recommended against an electroencephalogram (EEG) given his known history of seizures; a prior EEG was unremarkable. Computerized tomography (CT) of the head notably revealed a small frontal/lateral subdural hematoma measuring two millimeters that was not present during the previous hospitalization, which suggested possible head trauma associated with the seizure. A repeat CT head demonstrated no change in subdural hematoma size the following day. A urine drug screen was negative for amphetamines, cocaine, and other illicit drugs. Laboratory results showed the patient was negative for antinuclear antibodies, antimitochondrial antibodies, and anti-Jo-1 IgG antibodies. A CK level was obtained seven minutes after presentation to the ED (2,694 U/L; normal range: 22 to 198 U/L) to further evaluate if muscle breakdown was present in the setting of suspected generalized tonic-clonic seizure. The slight CK elevation was presumed to be secondary to seizure. Despite administering appropriate IV fluids and resuming LEV, the patient’s CK levels continued to rise, peaking at 47,078 U/L on Day 5 (Figure 2). The patient was diagnosed with rhabdomyolysis with concomitant acute kidney injury. A new adverse drug reaction to LEV was suspected to be the cause of this patient’s persistently rising CK levels and rhabdomyolysis. LEV was discontinued on Day 5, and the patient’s CK levels subsequently began to trend back down. The patient’s serum creatinine (sCr) peaked at 3.60 mg/dL (normal range: 0.7 to 1.2 mg/dL) on Day 2 before trending back within the normal range on Day 5. The patient was then discharged on lacosamide 100mg PO BID recommended by the consulting neurologist as an alternative anti-epileptic drug and scheduled to follow up with a neurologist two weeks later for epilepsy, as well as a primary care provider in one week to recheck CK levels; however, the patient was lost to follow-up.

## Discussion

LEV is a second-generation antiepileptic drug first approved in the 1990s for generalized seizures. Although the exact mechanism of action is unknown, previous studies have suggested LEV affects GABA receptor turnover, modulates calcium homeostasis, and binds to synaptic vesicle protein 2A (SV2A), modulating the release of neurotransmitters into the synaptic cleft [4]. In slow muscle fiber motor nerve terminals, SV2A is expressed to a lesser degree than in the central nervous system. In affected patients, LEV is thought to bind to bind to SV2A on motor nerve terminals, leading to cholinergic overstimulation, stress, and rhabdomyolysis [5]. Of note, LEV is an enantiomer of etiracetam, an acetylcholine agonist, which further supports the hypothesized mechanism of the over-stimulation of muscle cells [5]. However, LEV-induced rhabdomyolysis is rarely observed despite its widespread use; LEV has been implicated in at least 13 rhabdomyolysis cases based on our literature review [3]. Uniquely, there were no case reports or primary literature supporting LEV-induced rhabdomyolysis in a patient who has previously tolerated the medication. 

There were several reasons a new adverse drug reaction to LEV was suspected to be the cause of this patient’s persistently rising CK levels and rhabdomyolysis. First, his CK levels were elevated during his previous hospitalization prior to LEV administration. The steady decline in CK in spite of LEV administration suggests the patient was tolerating LEV and the elevated CK level was likely related to seizure and not a drug reaction at that time. Second, his CK levels peaked on the last day of LEV administration (day five) and steadily decreased after the discontinuation of LEV. This timeline closely mirrors the timeline and level of CK elevation of a previous case report by Di Lorenzo and Li, where CK levels peaked at 49,539 U/L within 48 hours of discontinuation of LEV and steadily trended downward [6]. Third, the patient remained asymptomatic except for diffuse muscle soreness on day five; seizure-induced rhabdomyolysis generally results in peak CK levels within the first 36 to 40 hours after the event [7]. Fourth, the only medication the patient received was LEV. Fifth, the negative results for antinuclear antibodies, antimitochondrial antibodies, and anti-Jo-1 IgG antibodies suggested the patient had no underlying myositis, myodystrophy, or autoimmune disorders commonly associated with rhabdomyolysis. Sixth, the patient did not have subjective or objective evidence of infection prior to or during admission [8]. Seventh, the half-life of LEV in adults with epilepsy is seven hours, which supports the down-trending CK levels in our patient after one day of discontinuation [9]. Lastly, the patient’s drug screen was negative for cocaine, amphetamines, and other illicit drugs known to precipitate rhabdomyolysis. 

While LEV-induced rhabdomyolysis is possibly explained by LEV binding to SV2A, the mechanism by which rhabdomyolysis occurred after the re-initiation of LEV remains unknown. Pharmacokinetic trials for LEV suggest the drug does not accumulate after multiple administrations and is metabolized into inactive metabolites via hydrolysis in the blood [10]. Therefore, it is unlikely that LEV accumulated in our patient and induced rhabdomyolysis despite one month of non-adherence. 

Though it is not the focus of this case report, it should be noted that acute kidney injury (AKI) due to LEV can be seen with and without evidence of rhabdomyolysis. There are several case reports describing both normal and high-loading doses of LEV associated with AKI without rhabdomyolysis, though it appears the peak serum creatinine often coincides with peak CK level temporally. Several reports also demonstrate a relationship between LEV and interstitial nephritis, though this type of kidney injury occurred much later after starting LEV compared to acute kidney injury which typically happens within one day of LEV initiation as seen in our case [11,12].

## Conclusions

Although LEV is generally well tolerated, previous studies have reported its potential to induce rhabdomyolysis. In this case report, we have shown that rhabdomyolysis can occur after re-initiation of LEV in a patient who had previously tolerated the medication. Clinicians should closely monitor serum CK and creatinine levels within the first several days of treatment initiation in those starting or restarting LEV. If renal function declines gradually, weeks after LEV initiation, interstitial nephritis secondary to LEV should be considered in the differential diagnosis; in the case of elevated CK or evidence of renal dysfunction, the medication should be discontinued in favor of an alternative anti-epileptic drug to rule out LEV as a cause. In addition, as this case report serves as the first reported incidence of LEV re-initiation reaction, it is conceivable that other drugs may cause the same phenomenon. Further drug monitoring and reporting of adverse effects are required if this phenomenon is to be better understood.
